# Factors associated with diagnostic and pre‐treatment intervals among breast cancer patients attending care at the Uganda Cancer Institute: A cross‐sectional study

**DOI:** 10.1002/cam4.6618

**Published:** 2023-10-03

**Authors:** Jennifer Achan, Francis Xavier Kasujja, Ronald Opito, Henry Wabinga, Christopher Garimoi Orach, Amos Deogratius Mwaka

**Affiliations:** ^1^ Department of Community Health & Behavioral Sciences, School of Public Health College of Health Sciences, Makerere University Kampala Uganda; ^2^ Department of Public Health, School of Health Sciences Soroti University Soroti Uganda; ^3^ Department of Pathology, School of Biomedical Sciences College of Health Sciences, Makerere University Kampala Uganda; ^4^ Department of Medicine, School of Medicine College of Health Sciences, Makerere University Kampala Uganda; ^5^ Department of Medicine, Faculty of Medicine Gulu University Gulu Uganda

**Keywords:** breast cancer, cancer stage, diagnosis, diagnostic interval, pre‐treatment interval

## Abstract

**Background:**

Most breast cancer (BC) patients in Uganda are diagnosed with advanced‐stage disease and experience poor outcomes. This study examined the diagnostic and pre‐treatment intervals and factors associated with these intervals among BC patients attending care at the Uganda Cancer Institute (UCI).

**Methods:**

This was a cross‐sectional, facility‐based study. Data were collected using structured questionnaire administered by trained research assistants and analyzed using STATA version 14.0. Modified Poisson regressions models were used to determine the strength of associations between independent variables and diagnostic and pre‐treatment intervals.

**Results:**

The mean age (±SD) of the 401 participants was 47.1 ± 11.7 years. Four in 10 participants had stage III (41.9%; *n* = 168) and over a third (34.7%; *n* = 140) stage IV cancers. The median interval from first consultation to diagnosis, i.e. diagnostic interval (DI) was 5.6 months (IQR: 1.5–17.0), while the median interval from histological diagnosis to start of chemotherapy, i.e. pre‐treatment interval (PTI) was 1.7 months (IQR: 0.7–4.5). Majority (85%, *n* = 341) of participants were diagnosed at ≥3 months from first consultation with clinicians. Participants with tertiary education and those who lived within 100–199 km from the UCI were about four times and twice more likely to be diagnosed early (DI <3 months from first consultation) ([aPR = 3.88; 95% CI: 1.15–13.0] and [aPR = 2.19; 95% CI: 1.06–4.55]), respectively. About half (48.3%; *n* = 176) of participants started chemotherapy within 1 month of cancer diagnosis. Patients who lived more than 300 km from the UCI were less likely to start chemotherapy within 1 month of histology diagnosis of cancer. [Correction added on October 17, 2023 after first online publication. The term ‘, i.e.‘ has been included in the results section in this version.]

**Conclusion:**

Majority of breast cancer patients are diagnosed late and in advanced stages. There is need to promote all efforts toward timely diagnosis when cancers are still in early stages by identifying factors responsible for prolonged diagnostic intervals among breast cancer patients.

## 1 BACKGROUND

The diagnostic interval (DI) is the period from when the patient first visits a primary healthcare provider to when he or she gets a confirmed diagnosis of cancer.^1^ Long diagnostic intervals among cancer patients have been associated with more advanced‐stage cancers at diagnosis, thus resulting in poor treatment outcomes including low chances for survival.^2^ Patients with symptoms of breast cancer who are diagnosed within 3 months of presentation are more likely to be at early stages and to have significantly better survival.^3^ An understanding of the factors that influence patients' journey to breast cancer diagnosis and treatment is vital for the development of targeted, context‐specific interventions to improve survival from breast cancer.^4^, ^5^, ^6^ In the United Kingdom (UK), primary care delays in diagnosis were found to contribute to a greater proportion of time from onset of symptoms to definitive diagnosis than delays in starting cancer treatment. Factors identified that seem to influence time taken to seek healthcare included patient factors (age, gender), health system factors (access, patient‐doctor communication), psychological factors (anxiety, fear), social factors (competing priorities), and behavioral factors (self‐medication, watchful waiting).^6^ In the low‐ and middle‐income countries (LMICs), delays in diagnosis of breast cancer have been attributed to low income, high transport costs to the cancer treatment facilities, high cost of in‐hospital care for patients and families, fear about diagnosis and treatment of cancers, low recognition of breast cancer symptoms, delay to initiate diagnostic tests by primary healthcare professionals, use of traditional medicines, and diagnostic errors.^7^, ^8^, ^9^, ^10^, ^11^ In sub‐Saharan Africa, a study of 2588 breast cancer patients from 14 population‐based cancer registries in 12 countries showed that majority of patients (64.9%) were diagnosed at advanced stages. Most patients experienced poor 3‐year relative survival ranging from 21.6% (95% CI: 8.2–39.8) in Bulawayo, Zimbabwe to 84.5% (95% CI: 70.6–93.5) in Namibia.^12^ In Uganda, more than 80% of women presenting for breast cancer treatment are in advanced disease stages. The long diagnostic intervals and advanced stages at diagnoses have been attributed to a dysfunctional referral system and lack of recognition of the early signs and symptoms among primary healthcare providers.^5^ In general, the total delay from presentations to initiation of cancer‐specific treatments is significantly associated with more advanced cancers at diagnoses.^13^, ^14^ Minimizing time to diagnosis is dependent on timely presentation to primary healthcare providers by patients with symptoms suggestive of breast cancer, appropriate assessment at the primary healthcare level, and timely access to referral and treatment centers.^15^ The costs of breast biopsies and pathologic examinations also lead to delayed diagnosis and advanced‐stage cancers at diagnoses.^16^ Clinical skills of primary healthcare professionals in suspecting and diagnosing or referring patients for diagnosis are critical to cancer early detection. Number of times a patient visited health facilities since onset of symptoms is a pointer to delay for diagnosis as the healthcare providers may not know the signs and symptoms of breast cancer and do not refer the patients to tertiary facilities for biopsy and diagnosis.^17^ Increased number of consultations with the surgeons before diagnosis^18^ and frequent visiting of the health facilities e.g., more than four times from symptom discovery are significantly associated with advanced‐stage breast cancer at diagnosis (over 86.4% were in stages III and IV).^4^ In Rwanda, it was shown that primary healthcare professionals entertained other diagnoses and delayed to refer patients to secondary and tertiary care facilities. In that study, frequent visit to the primary healthcare facilities before referral to the tertiary cancer facility significantly contributed to diagnostic delays and advanced‐stage breast cancers at diagnoses.^19^ However, in Uganda, there is limited data regarding diagnostic and pre‐treatment intervals of breast cancer patients. This study sought to examine the diagnostic and pre‐treatment intervals and the factors associated with them among patients with breast cancer attending care at Uganda Cancer Institute. Understanding these factors, especially health systems and patients' characteristics are important for informing targeted interventions to shorten time to diagnosis and start of cancer‐specific treatments.

## 2 METHODS

### 2.1 Study design and site

This cross‐sectional study was conducted at the Uganda Cancer Institute (UCI), a public specialized tertiary cancer center in Uganda that provides cancer‐specific and supportive treatments, research, and training in cancers. The UCI started in 1967 as a research collaboration between Makerere University, Mulago National Referral and Teaching hospital, and the United States National Cancer Institute.^20^ The Institute eventually evolved and assumed its current status as the oncology center of excellence for the East African region. Patients with all types of cancers are managed at the UCI. Furthermore, UCI provides services to cancer patients from the neighboring countries including Kenya, Tanzania, Rwanda, South Sudan, and the Democratic Republic of Congo (DRC).

### 2.2 Study population, sample size, and sampling procedure

Leslie Kish (1965) formula for cross‐sectional studies was used to calculate sample size based on assumption that 50% of BC patients were diagnosed before 3 months from first consultations and started chemotherapy within 1 month of cancer diagnosis. Precision of 5% was allowed. The estimated sample size at 95% confidence interval (CI) = 384; we increased the sample size by 10% to allow for nonresponse/missing data, and hence total sample size is 423 (384 + 38.4) participants.

All adult (aged ≥18 years) Ugandan patients with histology diagnosis of breast cancer diagnosed within 24 months of study onset, attending care at UCI during the study period, and undergoing adjuvant chemotherapy were consecutively sampled. It was assumed that patients diagnosed within 24 months could still have reasonable recall of dates of events including dates of symptoms onset and first health‐seeking. Limiting the dates of diagnoses to within recent 24 months also helped to avoid selection bias through effect of survivorship. We excluded non‐Ugandan breast cancer patients who may have different pathways to treatment because of several other country‐specific factors. Asymptomatic patients diagnosed through screening, patients with incomplete medical records e.g., lacking referral forms and histology reports, and very ill patients were also excluded from this study.

Three female research assistants were trained on the study purpose, objectives, and procedures including consenting and data collection and storage. The research assistants included two graduates of social sciences who did not work at the UCI and a nursing officer from the UCI who helped in identification of patients' files for purposes of data extraction. The study team approached breast cancer patients as they registered and waited for consultations and or treatments at the outpatients clinic. All stable breast cancer patients admitted on the wards were also approached and requested to participate in the study.

### 2.3 Data collection

Data collection was conducted between June and October 2019 after approval of the study protocol. Trained Research Assistants (RA) collected data using pre‐tested, semi‐structured questionnaire (Data S1), adapted from the African Women Awareness of Cancer (AWACAN) tool and the Model of Pathways to Treatment (MPT).^6^, ^21^ The questionnaire included information on: sociodemographic profile of participants, awareness of breast cancer risk factors and symptoms, cancer symptoms experienced, health seeking, and dates of key events on the pathway to care. The tool also had questions on health system factors including number of times patients visited the health facilities before referral and visit to the UCI, distance from patient's home to nearest health facility, distance from home to the UCI, and disease factors including cancer stage and tumor differentiations. The study tool was pre‐tested and refined on the basis of the data from the pre‐test. The 10 patients included in the pre‐test were excluded from the main study.

The research assistants (RAs) provided detailed information about the study objectives, selection procedures, and the rights of participants while in the study including the right to decline participations or withdraw at any time without fear of any negative consequences on their treatments at the UCI. The RAs administered the consent form before conducting the interviews in quiet rooms with privacy and minimal interference from non‐participants. The RAs were supervised by JA who also participated in data collection. Each interview lasted about 30–45 minutes. Data were collected using the Open Data Kit (ODK) software installed on android phones. Recall bias during interviews were minimized by use of calendar landmark approach, i.e., using prompts based on key events including Christmas day and the Independence Day.^22^ After interviews with the participants, the RAs used the patient's file numbers linked to the participants' identification numbers to identify the patients' case notes from records to extract data on tumor characteristics including stage at diagnosis and date of initiation of chemotherapy. Data abstracted from the patients' files included patients' date of histology diagnosis, date of referral to UCI, cancer stage at diagnosis, and date of start of chemotherapy. The RAs downloaded data from their android phones at the end of every day's work onto the investigator's laptop that was secured with a password for confidentiality of data. Data collection continued on the clinic days and every day on the wards, until the sample size was achieved.

### 2.4 Data management and analysis

JA reviewed data with research assistants at the end of every day of data collection and ensured that the patients' file numbers and study numbers were appropriately matched, and checked contents for completeness. Questionnaires with incomplete data were filled by revisiting the patients and or the patients' files. If the patient was an outpatient, then they were met in their next visits as per the schedules in the patient's file. This was done to ensure completeness, consistency, and accuracy of data before storage. Data were downloaded from the ODK in Excel format. Data cleaning and editing were conducted by JA and RO. RO exported data from excel to STATA version14.0 for further cleaning, coding, and analysis. New variables were created to conform to the study dependent variables. The key outcomes in the analysis were diagnostic interval (DI) i.e., date of histology diagnosis minus date of first consultation with healthcare professionals (HCP), and pretreatment interval (PTI) i.e., date of start of adjuvant chemotherapy at the UCI minus date of histological diagnosis of breast cancer. These dates were selected to conform to the international standard criteria for determinations of intervals in the pathway to diagnosis and treatment of cancers.^1^, ^6^, ^23^ The study population was described using proportions for categorical variables, while continuous variables were summarized using medians and interquartile ranges and mean and standard deviations. Chi‐square tests were used to examine associations between sociodemographic characteristics and health systems factors, and the study outcomes including diagnostic and pre‐treatment intervals. Acceptable diagnostic intervals for breast cancer are at 3 months, because delays of 3–6 or more months lead to poorer survival.^24^ Accepted pre‐treatment interval for starting adjuvant chemotherapy for breast cancer is 1–2 months.^25^, ^26^, ^27^ A study by Kumar^28^ involving 332,927 breast cancer patients on time to start adjuvant chemotherapy among patients diagnosed from 2010 to 2016 showed optimal time to be 31–60 days. In this study, 1 month was used as cutoff for timely initiation of chemotherapy.

Multivariable analyses to establish factors associated with diagnostic and pre‐treatment intervals were conducted using modified Poisson regression with robust variance which provides a direct and consistent estimate of the relative risk (effect size) with greater accuracy than logistic regressions.^29^ Variables to include in the regression models were identified apriori based on clinical relevance. The regression was modeled to provide outputs as follows: diagnostic intervals less than 3 months (favorable outcome) was assigned “Yes” i.e., =1 and 0 otherwise; and for the pre‐treatment intervals, an interval less than 1 month (favorable outcome) was assigned “Yes” i.e., =1 and 0 otherwise. Therefore, adjusted prevalence ratio (aPR) >1 denotes early diagnosis and prompt onset of adjuvant chemotherapy (i.e., within <1 month). Prevalence ratios with their corresponding 95% confidence intervals have been reported. Prevalence ratio is easier to directly interpret than odds ratios in cross‐sectional studies with binary outcomes.^30^

Prevalence ratios with their corresponding 95% confidence intervals have been reported. Statistical significance was considered if associated two‐sided *p*‐value of a PR is <0.05.

## 3 RESULTS

### 3.1 Sociodemographic characteristics of study participants

A total of 423 participants were recruited; of these, 401 participants had complete data necessary for answering the study objectives. Table 1 shows the sociodemographic characteristics of the participants; the majority were female (96.5%; *n* = 387). The mean age was 47.1 ± 11.7 years. Majority of participants were in either stage III (41.9%; *n* = 168) or stage IV (34.7%; *n* = 140) cancers.

**TABLE 1:** Participants' sociodemographic characteristics.

Characteristics	Population (*N*)	Percentage (%)
Sex		
Male	14	3.5
Female	387	96.5
Marital status		
Single	26	6.4
Married/stay with partner	286	71.3
Divorced/separated	41	10.2
Widower	48	11.9
Age group (years)		
<40	104	25.9
40–49	139	34.7
50–59	100	24.9
≥60	58	14.4
Education		
No formal education	45	11.2
Primary	142	35.4
Secondary	106	26.4
Tertiary	108	26.9
Region		
Central	190	47.4
Eastern	75	18.7
Northern	71	17.7
Western	65	16.2
Employment status		
No formal employment	237	59.1
Employed	164	40.9
Family history of breast cancer		
No	245	61.1
Yes	99	24.7
Do not know	57	14.2
Breast cancer stage		
I	15	3.7
II	79	19.7
III	168	41.9
IV	140	34.7
Parity		
<5 children	238	59.4
≥5 children	149	37.1
Not applicable (i.e., men)	14	3.5

### 3.2 Diagnostic and pre‐treatment intervals

The median time from first formal consultation to histology diagnosis (diagnostic interval; DI) and the median time taken from histology diagnosis to start of adjuvant chemotherapy (pre‐treatment interval; PTI) are shown in Table 2.

**TABLE 2:** Diagnostic and pre‐treatment intervals.

Intervals	Median (months)	Interquartile range (months)
Diagnostic interval (DI)		
Time from 1st consultation to diagnosis	5.6	1.5–17.0
Pre‐treatment intervals (PTI)		
Time from diagnosis to treatment	1.7	0.7–4.5

### 3.3 Factors associated with diagnostic intervals

#### 3.3.1 Patients sociodemographic factors

Table 3 shows the sociodemographic characteristics in relation to diagnostic intervals; 15% (*n* = 60) were diagnosed by 3 months after first formal consultation (early diagnosis). Participants with tertiary education were about four times more likely to be diagnosed early (before 3 months of first formal consultation), adjusted PR [aPR] = 3.88 (95% CI: 1.15–13.0 (Table 3). [Correction added on October 17, 2023 after first online publication. Punctuation has been adjusted in the preceding sentence.]

**TABLE 3:** Patient sociodemographic factors and diagnostic intervals.

Factors considered	Diagnostic interval (DI)		Unadjusted PR (95% CI)	Adjusted PR (95% CI)	*p*‐Value
	DI <3 months *N* (%)	DI ≥3 months *N* (%)			
Marital status					
Single	2 (7.7)	24 (92.3)	1.00	1.00	
Married	41 (14.3)	245 (85.7)	1.86 (0.48–7.28)	1.71 (0.47–6.13)	0.41
Divorced	7 (17.1)	34 (83.0)	2.22 (0.50–9.89)	1.73 (0.41–7.22)	0.46
Widow	10 (20.8)	38 (79.2)	2.71 (0.64–11.47)	3.16 (0.75–13.36)	0.12
Age group (years)					
<40	11 (10.6)	93 (89.4)	1.00	1.00	
40–49	22 (15.8)	117 (84.2)	1.50 (0.76–2.95)	1.45 (0.75–2.80)	0.27
50–59	18 (18.0)	82 (82.0)	1.70 (0.85–3.42)	1.71 (0.82–3.82)	0.15
≥60	9 (15.5)	49 (84.5)	1.47 (0.65–3.33)	1.51 (0.57–3.96)	0.41
Education level					
No formal education	3 (6.7)	42 (93.3)	1.00	1.00	
Primary	18 (12.7)	124 (87.3)	1.90 (0.59–6.17)	1.63 (0.50–5.31)	0.42
Secondary	15 (14.2)	91 (85.9)	2.12 (0.0.65–6.98)	2.12 (0.62–7.27)	0.23
Tertiary	24 (22.2)	84 (77.8)	**3.33 (1.06–10.53)**	**3.88 (1.15–13.04)**	**0.03**
Employment status					
No formal employment	34 (14.4)	203 (85.7)	1.00	1.00	
Employed	26 (15.9)	138 (84.2)	1.11 (0.69–1.77)	0.75 (0.45–1.26)	0.28
Region of origin in Uganda					
Central	31 (16.3)	159 (83.7)	1.00	1.00	
Eastern	12 (16.0)	63 (84.0)	0.98 (0.53–1.81)	1.10 (0.59–2.03)	0.77
Northern	6 (8.5)	65 (91.6)	0.52 (0.23–1.19)	0.56 (0.25–1.25)	0.16
Western	11 (16.9)	54 (83.1)	1.04 (0.55–1.94)	1.33 (0.71–2.47)	0.38
Family history of BC					
No	33 (13.5)	212 (86.5)	1.00	1.00	
Yes	20 (20.2)	20 (20.2)	1.50 (0.91–2.48)	1.20 (0.73–1.10)	0.47
Do not know	7 (12.3)	50 (87.7)	0.91 (0.42–1.96)	1.03 (0.48–2.22)	0.94
Having prior information on BC					
No	5 (6.1)	77 (93.9)	1.00	1.00	
Yes	55 (17.3)	264 (82.8)	**2.83 (1.17–6.84)**	2.44 (0.95–6.24)	0.06
Risk factor knowledge level					
Low	42 (16.7)	210 (83.3)	1.00	1.00	
High	18 (12.1)	131 (87.9)	0.72 (0.43–1.21)	0.72 (0.43–1.19)	0.20

*Note*: Bold = statistically significant with *p* < 0.05; BC = breast cancer; PR = prevalence ratio; all factors in the table were adjusted for each other. aPR >1 denotes early diagnosis (within 3 months).

#### 3.3.2 Association between health system factors and diagnostic intervals

The only health system factor significantly associated with diagnostic intervals was distance from home to the UCI (in kilometers). Patients who resided within 100–199 km from the UCI were twice more likely to have been diagnosed before 3 months from first formal consultations (Table 4).

**TABLE 4:** Health system factors and diagnostic intervals (DI).

Factors considered	Diagnostic interval (DI)		Unadjusted PR (95% CI)	Adjusted PR (95% CI)	*p*‐Value
	DI <3 months *N* (%)	DI ≥3 months *N* (%)			
Distance from home to nearest HF (KM)					
<5	30 (13.2)	197 (86.8)	1.00	1.00	
5–15	21 (17.8)	97 (82.2)	1.35 (0.81–2.25)	1.45 (0.86–2.43)	0.16
>15	9 (16.1)	47 (83.9)	1.22 (0.61–2.41)	1.34 (0.65–2.78)	0.43
Distance from home to UCI (Kilometers)					
<5	8 (16.7)	40 (83.3)	1.00	1.00	
5–99	16 (11.9)	118 (88.1)	0.72 (0.33–1.57)	0.72 (0.34–1.51)	0.38
100–199	16 (32.7)	33 (67.4)	1.96 (0.92–4.15)	**2.19 (1.06–4.55)**	**0.04**
200–299	7 (9.7)	65 (90.3)	0.58 (0.23–1.50)	0.67 (0.24–1.83)	0.43
300–399	8 (16.7)	40 (83.3)	0.1 (0.41–2.45)	1.64 (0.56–4.76)	0.37
>400	5 (10.0)	45 (90.0)	0.6 (0.21–1.71)	1.97 (0.27–3.45)	0.80
Number of visits to HF before referral to UCI					
Once	26 (16.5)	132 (83.5)	1.00	1.00	
Two times	15 (15.2)	84 (84.9)	0.92 (0.51–1.65)	0.97 (0.54–1.74)	0.91
Three times	16 (15.5)	87 (84.5)	0.94 (0.53–1.67)	0.99 (0.56–1.76)	0.98
≥Three times	3 (7.3)	38 (92.7)	0.44 (0.14–1.40)	0.50 (0.17–1.51)	0.22
Level of referring HF					
Self‐referral	13 (12.9)	88 (87.1)	1.00	1.00	
Private clinic	6 (16.7)	30 (83.3)	1.29 (0.53–3.15)	1.44 (0.60–3.50)	0.42
Level 4 (HCIV)	1 (7.1)	13 (92.9)	0.55 (0.09–3.93)	0.68 (0.10–4.43)	0.68
Level 5 (GH)	9 (20.5)	35 (79.6)	1.59 (0.73–3.44)	1.91 (0.85–4.32)	0.12
Level 6 (RRH)	14 (14.0)	86 (86.0)	0.09 (0.54–2.20)	1.42 (0.64–3.18)	0.39
Level 7 (NRH)	17 (16.0)	89 (84.0)	1.25 (0.64–2.43)	1.29 (0.64–2.62)	0.47

*Note*: HF = health facility; HCIV = health center IV; RRH = Regional Referral Hospital; NRH = National Referral hospital; factors adjusted for: age, marital status, employment, education level, and region. aPR >1 denotes early diagnosis. Bold = Statistically significant factors.

### 3.4 Factors associated with pre‐treatment intervals

Of the 401 participants who were diagnosed with BC, 8.2% (*n* = 33) had not yet started adjuvant chemotherapy by the time of data collection and were excluded from further analysis of pre‐treatment intervals. Of the 368 participants who had started treatment, 47.8% (*n* = 176) were initiated on chemotherapy promptly, (PTI ≤ 1.0), while 52.2% (*n* = 192) were initiated late (PTI > 1) (Table 5). [Correction added on October 17, 2023 after first online publication. Punctuation has been adjusted in the preceding sentence.]

**TABLE 5:** Patient factors and pre‐treatment intervals (PI).

Factors considered	Pre‐treatment Interval (PI)		Unadjusted PR	Adjusted PR	*p*‐Value
	PI <1 month *N* (%)	PI ≥1 month *N* (%)			
Marital status					
Single	8 (33.3)	16 (66.7)	1.00	1.00	
Married/stay with partner	76 (29.0)	186 (71.0)	0.87 (0.48–1.58)	0.77 (0.42–1.44)	0.42
Divorced/separated	13 (35.1)	24 (64.9)	1.05 (0.51–2.16)	0.98 (0.45–2.14)	0.95
Widow	15 (33.3)	30 (66.7)	1 (0.50–2.02)	1.10 (0.52–2.34)	0.80
Age group (years)					
<40	30 (30.9)	67 (69.1)	100	100	
40–49	39 (30.2)	90 (69.8)	0.98 (0.66–1.45)	1.02 (0.66–1.56)	0.93
50–59	26 (29.6)	62 (70.5)	0.96 (0.62–1.48)	0.87 (0.50–1.51)	0.61
≥60	17 (31.5)	37 (68.5)	1.02 (0.62–1.67)	0.81 (0.42–1.55)	0.52
Education level					
No formal education	14 (34.2)	27 (65.9)	1.00	1.00	
Primary	38 (29.5)	91 (70.5)	0.86 (0.52–1.43)	0.74 (0.43–1.26)	0.27
Secondary	26 (26.8)	71 (73.2)	0.78 (0.46–1.34)	0.68 (0.38–1.20)	0.18
Tertiary	34 (33.7)	67 (66.3)	0.99 (0.60–1.64)	0.83 (0.46–1.50)	0.54
Employment status					
No formal employment	61 (28.4)	154 (71.6)	1.00	1.00	
Employed	51 (33.3)	102 (66.7)	1.17 (0.86–1.60)	1.31 (0.91–1.90)	0.15
Region of origin in Uganda					
Central	57 (33.3)	114 (66.7)	1.00	1.00	
Eastern	25 (36.2)	44 (63.8)	1.09 (0.74–1.59)	1.12 (0.76–1.64)	0.58
Northern	11 (16.7)	55 (83.3)	**0.50 (0.28–0.89)**	**0.48 (0.27–0.87)**	**0.02**
Western	19 (30.7)	43 (69.4)	0.92 (0.60–1.41)	0.90 (0.57–1.43)	0.66
Family history of breast cancer					
No	68 (30.9)	152 (69.1)	1.00	1.00	
Yes	30 (32.3)	63 (67.7)	1.04 (0.73–1.49)	0.98 (0.68–1.43)	0.92
Do not know	14 (25.5)	41 (74.6)	0.82 (0.50–1.35)	0.80 (0.47–1.36)	0.41
Age at starting menstruation (years)					
<15	64 (30.8)	144 (69.2)	1.00	1.00	
>15	43 (29.3)	104 (70.8)	0.95 (0.69–1.31)	1.02 (0.73–1.43)	0.90
Postmenopausal					
No	38 (32.8)	78 (67.2)	100	100	
Yes	69 (28.9)	170 (71.1)	0.88 (0.63–1.22)	1.01 (0.66–1.54)	0.98

*Note*: Bold = statistically significant factors. PR = prevalence ratio, PI = pre‐treatment interval. All factors on the table were adjusted for each other. aPR >1 denotes prompt onset of adjuvant chemotherapy (i.e., within 1 month).

#### 3.4.1 Patient sociodemographic factors and pre‐treatment intervals

There were no statistically significant associations between patient sociodemographic factors (except for region of origin) and the pre‐treatment intervals. Patients coming from the northern region were less likely to start treatment within 1 month from histologic diagnosis (aPR = 0.48; 95% CI: 0.27–0.87), as compared to those who came from the central region (Table 5).

#### 3.4.2 Health system factors and pre‐treatment intervals

More than a quarter, (26.4%; *n* = 106) of participants were referred from national referral hospitals, 25.2% (*n* = 101) were self‐referral, and 24.9% (*n* = 100) were from regional referral hospitals. The median distance from the participants' homes to the UCI was 150 km (IQR 16–305) while the median distance from patient's home to nearest health facility was 4 km (IQR, 2–7). There were no statistically significant associations between any of the health system factors examined and pre‐treatment intervals (Table 6).

**TABLE 6:** Health system factors and pre‐treatment intervals (PI).

Factors considered	Pre‐treatment interval (PI)		Unadjusted PR	Adjusted PR	*p*‐Value
	PI <1 months *N* (%)	PI ≥1 months *N* (%)			
Distance from home to nearest HF (Kilometers)					
<5	72 (33.8)	141 (66.2)	1.00	1.00	
5–<15	28 (26.2)	79 (73.8)	0.77 (0.53–1.12)	1.45 (0.86–2.43)	0.16
>15	12 (25.0)	36 (75.0)	0.74 (0.45–1.25)	1.34 (0.65–2.78)	0.43
Distance from home to UCI (KM)					
<5	13 (30.2)	30 (69.8)	1.00	1.00	
5–99	42 (34.7)	79 (65.3)	1.15 (0.69–1.92)	1.12 (0.65–1.90)	0.69
100–199	13 (27.7)	34 (72.3)	0.91 (0.48–1.75)	0.85 (0.42–1.73)	0.66
200–299	29 (41.4)	41 (58.6)	1.37 (0.80–2.34)	1.40 (0.73–2.69)	0.31
300–399	7 (17.1)	34 (82.9)	0.56 (0.25–1.27)	0.65 (0.25–1.67)	0.37
>400	8 (17.4)	38 (82.6)	0.58 (0.26–1.25)	0.72 (0.30–1.78)	0.49
Time from referral to first visit to the UCI (months)					
<1	28 (27.5)	74 (72.6)	1.00	1.00	
1–<3	33 (29.7)	78 (70.3)	0.77 (0.40–1.49)	1.13 (0.73–1.74)	0.59
3–<6	10 (30.3)	23 (69.7)	0.78 (0.38–1.58)	1.11 (0.60–2.06)	0.74
>6	41 (33.6)	81 (66.4)	0.61 (0.29–1.27)	1.23 (0.82–1.84)	0.32
Number of visits to health facilities before referral to UCI					
Once	49 (34.3)	94 (65.7)	1.00	1.00	
Two times	29 (31.2)	63 (68.5)	0.92 (0.51–1.65)	0.88 (0.60–1.30)	0.52
Three times	28 (29.2)	68 (70.8)	0.94 (0.53–1.67)	0.79 (0.53–1.17)	0.24
Above three times	6 (16.2)	31 (83.8)	0.44 (0.14–1.40)	0.47 (0.22–1.02)	0.06

*Note*: PR = prevalence ratio. Adjustments done for age, marital status, education level, employment, and region. UCI = Uganda Cancer Institute, PI = pre‐treatment interval. aPR >1 denotes prompt onset of adjuvant chemotherapy (i.e., within 1 month).

## 4 DISCUSSION

In this study, most participants (85%) took long to receive histology diagnoses of breast cancer and were diagnosed with advanced‐stage cancer. Our findings are similar to results from Rwanda where the median time from first consultation to diagnosis was 5 months, and majority of patients were in advanced stages of the disease.^19^ Similarly, long time to diagnosis of breast cancer was reported in Malaysia where the median time to receive histology diagnosis was 5.5 months.^31^ In a multicountry study involving 1429 breast cancer patients, the median diagnostic time in Uganda was 11.7 months (IQR: 5.7–21.2) and 8.2 months (IQR:3.4–16.4) in Zambia.^32^ Therefore, majority of patients with breast cancer symptoms in most low‐ and middle‐income countries receive breast cancer histological diagnoses late. However, our finding of long diagnostic interval differs from findings in Mali and South Africa, as well as in Mexico. In Mali, the diagnostic interval was shorter, with median diagnostic interval of 0.9 months. The short diagnostic interval in Mali was attributed to adequate knowledge of breast self‐examination and correct symptom interpretations by primary healthcare professionals that promoted prompt detection of breast cancers.^33^ In South Africa, a study showed a median diagnostic interval of 28 days compared to our findings of 5.6 months.^4^ In Mexico, a study in four centers showed that majority of breast cancer patients were diagnosed in advanced‐stage disease and had a median diagnostic interval of 4 months.^14^ In the high‐income countries, including the USA and UK, symptomatic women receive histology diagnoses of breast cancers within a shorter time from first consultations, and majority are diagnosed at early stages.^34^ In Canada, a study that assessed breast cancer patients diagnosed during 2007 showed that the median diagnostic interval was 36 days.^35^ And in Germany, most breast cancer patients diagnosed between 1996 and 1998 received cancer diagnoses within 15 days of first presentation to a physician. The diagnostic interval was associated with high education level, full‐time employment and family history of breast cancer.^36^ A review study showed that median total diagnostic interval for breast cancer is between 30 and 48 days in the high‐income countries and 3–8 months in the LMICs.^37^ Therefore, context‐relevant interventions to reduce time to diagnosis of breast cancer including attitude change, diagnostic service improvement, and improving the referral system of the health sector in the low‐ and middle‐income countries are crucial to promote early presentations and diagnosis. Better knowledge of cancer symptoms has been shown to help people recognize cancer symptoms early and therefore reduce the appraisal interval and time to diagnosis.^32^, ^33^, ^38^ Similarly, interventions to improve recognitions of breast cancer symptoms and signs by the primary healthcare professionals could promote prompt diagnosis and down‐staging of breast cancers among women in sub‐Saharan Africa and other low‐ and middle‐income countries. There is evidence that in the LMICs, significant proportions of patients with cancer symptoms visit lower‐level healthcare facilities several times, get misdiagnosed, and receive treatments for other conditions before referral for cancer diagnosis because of several reasons including low ability of the primary healthcare professionals to suspect cancers.^39^, ^40^

This study showed that participants with tertiary education were about four times more likely to be diagnosed within 3 months as compared to those with no formal education (aPR = 3.88; 95% CI: 1.15–13.0). Another study in sub‐Saharan Africa also showed statistically significant associations between diagnostic intervals and patient factors including age, marital status, and employment.^41^ In Canada, a study involving 45,967 women diagnosed with breast cancer during 2007–2015 showed that longer diagnostic intervals were significantly associated with younger age and several visits to physicians prior to breast cancer diagnosis.^42^ Similarly, in Mexico, a study of 592 women managed with breast cancers in two public hospitals showed long diagnostic intervals were associated with younger age (i.e., 40 years or less) and advanced stage cancers at diagnoses.^43^ Younger women probably have low self‐perceived risks of breast cancer. Therefore, targeted interventions are needed to increase awareness of younger women (aged <40 years) of their risk to breast cancer and increased risk of being diagnosed with advanced‐stage breast cancer.

We also found that the only health system factor associated with the diagnostic intervals was distance from home to the Uganda Cancer Institute (UCI). Participants who resided within 100–199 km from UCI were less likely to be diagnosed late as compared to those who stayed less than 5 km from the UCI. A plot of the prevalence ratios (results not shown) against the distances from the UCI approximates a U‐shape curve. The UCI does not actively participate in cancer diagnosis except through providing screening services. The UCI usually receives patients with established histology diagnosis of cancers and provides chemotherapy, radiotherapy, surgery, and other supportive treatments. The diagnostic dynamics and intervals within the capital city where UCI is situated may be influenced by several factors including services costs and economic status, psycho‐social factors, and attitudinal concerns regarding cancer and cancer services at the UCI. Similar findings of U‐shaped associations in regards to distance from treatment facility, and cancer stage as well as 5‐year mortality have been reported before, especially for colorectal and oral cancers.^44^, ^45^, ^46^, ^47^, ^48^, ^49^ Regarding distance from cancer treatment facilities and mode of treatment, breast cancer patients in the USA living within 5 miles had lower odds of mastectomy compared to those living ≥40 miles.^50^ It is not completely understood why patients from near cancer‐specialized treatment facilities get diagnosed after longer time or choose a different modality of treatment compared with those living far. The people living so very close may know the deficiencies of their health system and or the challenges associated with receiving care at the facilities and would perhaps only go there as last resort, while those far away may have limited knowledge of the services and would endeavor to reach as soon as they can. We could not delineate these issues in the current study. Perhaps a qualitative study aimed at both delayers and prompt health seekers, stratified by the distances of their homes from the treatment facility, may help answer more accurately this important question.

There was no association between the diagnostic intervals and health system factors including distance from participants' home to nearest health facility, and number of visits to primary healthcare facilities by the participants. However, findings from other studies showed that frequent visits to the primary healthcare facilities before referral for cancer diagnosis contributed to delayed diagnosis of breast cancer.^4^, ^18^, ^19^, ^51^ The several visits to primary healthcare facilities before cancer diagnoses have been attributed to poor recognition of cancer symptoms and signs by the primary healthcare professionals who make other diagnoses and keep treating non‐cancer conditions as the cancers progress.^43^ In‐service training of primary healthcare professionals to improve their awareness and recognition of cancer symptoms and signs, as well as increase their clinical acumens, is required and has been shown to increase the chances of early cancer detection by the primary healthcare professionals, downstage cancers, and reduce the number of visits to primary healthcare facilities.^52^ LMICs may adopt in‐service trainings as one of the measures to improve cancer diagnosis and outcomes.

The median time from histological diagnosis to start of adjuvant chemotherapy (pre‐treatment interval) was 1.7 months, and this was generally within the acceptable one to 2 months required for cancer staging, healing of surgical wounds for patients who may have had surgical interventions, and preparation of patients for chemotherapy. However, only about half of participants started adjuvant chemotherapy within 1 month of histology diagnosis, and other participants experienced treatment delays for various reasons. The median pre‐treatment interval in this study is comparable to findings from other studies in sub‐Saharan Africa. In South Africa, a study showed median pre‐treatment interval of 37 days (1.2 months), while in Mali, a study showed a median pre‐treatment interval of 1.3 months.^4^, ^53^ The median pre‐treatment interval from this study is also comparable to results of a study from New Zealand, an upper‐middle‐income country where 59.6% of participants diagnosed with breast cancer started cancer‐specific treatments within 31 days (1 month) of diagnosis and 98% within 90 days.^54^ During the pre‐treatment intervals, patients undergo preparation for chemotherapy including staging and laboratory investigations of various systems. Although there are limited data from randomized control trials to show a particular median pre‐treatment interval with survival benefits, observational studies show that a pre‐treatment period of 1–2 months to prepare the patients for cancer‐specific treatment is considered acceptable.^25^, ^26^, ^27^ The optimal time for starting adjuvant chemotherapy for breast cancer patients who have undergone breast surgery is 31–60 months for all subtypes of breast cancer.^28^ Delaying adjuvant chemotherapy to beyond 90 days portends poor outcome.^55^

## 5 LIMITATIONS

This study had some limitations; it was a cross‐sectional study from which causal associations cannot be drawn between the sociodemographic and health systems' factors and the outcome variables including diagnostic and pre‐treatment intervals. Second, our findings could be influenced by recall bias since participants retrospectively recalled key dates for the assessment of outcomes—the diagnostic and pre‐treatment intervals. However, difficulty in recall and possible associated bias was minimized through the use of calendar landmark approach in which participants were prompted to recall by use of key events including Independence Day, important holidays, and religious events such as Christmas Day. We also limited recruitment to patients diagnosed no more than 24 months prior to recruitment. Third, the study setting was a tertiary‐level facility, and patients reaching this facility could be systematically different from those that have not managed to reach the facility; thus, caution needs to be taken when interpreting and generalizing findings from this study. However, the inclusion of participants from all over the country also means that data from the study can be appropriately used to inform national policies on cancer early detection and prompt treatment. Fourth, we used date of histology report as date of cancer diagnosis, and this has potential to underestimate the diagnostic interval compared to when date of biopsy is used. This, however, does not change the direction of the effect measures we have reported. Perhaps future studies could use date of biopsy to determine diagnostic intervals and then compare the overall findings with our approach of using date of histology.

## 6 CONCLUSIONS

Symptomatic breast cancer patients in Uganda receive confirmatory cancer diagnoses after several months from first consultations with primary healthcare professionals; more educated patients were more likely to be diagnosed early. Patients from far away from Uganda Cancer Institute had problems starting treatment after diagnosis. Apart from the above factors, qualitative studies among breast cancer patients and caregivers could explain other factors leading to prolong diagnostic and pre‐treatment intervals.

## AUTHOR CONTRIBUTIONS

**Jennifer Achan:** Conceptualization (equal); data curation (equal); investigation (equal); methodology (equal); project administration (equal); supervision (equal); validation (equal); visualization (equal); writing – original draft (equal). **Francis Xavier Kasujja:** Conceptualization (equal); investigation (equal); methodology (equal); project administration (equal); supervision (equal); validation (equal); visualization (equal); writing – review and editing (equal). **Ronald Opito:** Data curation (equal); formal analysis (equal); investigation (equal); methodology (equal); writing – review and editing (equal). **Henry Wabinga:** Conceptualization (equal); funding acquisition (equal); methodology (equal); project administration (equal); supervision (equal); validation (equal); visualization (equal); writing – review and editing (equal). **Christopher Garimoi Orach:** Conceptualization (equal); funding acquisition (equal); investigation (equal); methodology (equal); project administration (equal); supervision (equal); validation (equal); visualization (equal); writing – review and editing (equal). **Amos Deogratius Mwaka:** Conceptualization (equal); formal analysis (equal); funding acquisition (equal); methodology (equal); resources (equal); software (equal); supervision (equal); validation (equal); visualization (equal); writing – original draft (equal); writing – review and editing (equal).

## FUNDING INFORMATION

This work was funded through a student support to JA by a multinational research collaboration between Makerere University, University of Cape Town and University of Cambridge which was jointly supported by the Cancer Association of South Africa (CANSA), the University of Cape Town and the South African Medical Research Council with funds received from the South African National Department of Health, GlaxoSmithKline (GSK) Africa Non‐Communicable Disease Open Lab (via a supporting grant Project Number: 023), the United Kingdom Medical Research Council, MRC (via the Newton Fund). GSK provided in‐kind scientific and statistical support as part of capacity strengthening. Award/Grant number is not applicable. The funders had no role in study design, data collection and analysis, preparation of the manuscript, decision to publish, and where to publish.

## CONFLICT OF INTEREST STATEMENT

The authors declare that they have no competing interests.

## ETHICS STATEMENT

This study protocol was approved by the Makerere University School of Public Health Higher Degrees Research & Ethics Committee. Administrative clearance was obtained from the Executive Director of Uganda Cancer Institute. The research team with earlier training on ethics for research involving human subject recruited participants and collected data. The research assistants provided adequate information about the study and procedures to the participants before recruitment. These included information on purpose of the study, intended benefits, participants' rights during research including the right to decline participations and or withdraw at any time, and the confidentiality of their information during and after the interviews.

## INFORMED CONSENT

All study participants provided written informed consent prior to participation in the interviews. Privacy and confidentiality were observed during the data collection process by conducting the interviews one at a time in private rooms without non‐participants. Data collection tools were designed in English and translated into two local languages – Luganda and Acoli/Luo. A transport refund of USD 3.5 was provided to every participants after the interviews. The study used an online data collection system (ODK), and this was password encrypted to ensure limited access to the clients' information only to the research team. The information collected was de‐identified before analysis by dropping off identifiers such as registration number, clients' names, and address.

## Supporting information


Data S1.

## Data Availability

The datasets used and/or analyzed during the current study are available from the corresponding author on reasonable request.
