# Supplementary material for: Factors associated with diagnostic and pre‐treatment intervals among breast cancer patients attending care at the Uganda Cancer Institute: A cross‐sectional study
Source: Cancer Med. 2023 Oct 3;12(19):19701–13. doi: 10.1002/cam4.6618 (PMC10587984; doi:10.1002/cam4.6618)
Supplement: Supplementary file 1 — Data S1. [file CAM4-12-19701-s001.docx]

## **Supplementary File 1: Semi-structured questionnaire (English Version)**

| **Study Number (Linked with unique patient identifier)…………….** | | | | |
| --- | --- | --- | --- | --- |
| **SECTION 1: SOCIO-DEMOGRAPHIC CHARACTERISTICS** | | | | |
| 1 | Age in completed years | | |  |
| 2 | Gender | | | 1. Male  2. female |
| 3 | Tribe? | | |  |
| 4 | Current residence. | District | |  |
|  |  | Village | |  |
| 5 | Estimated distance from UCI (**to be filled afterwards using Google distance estimator**) | | |  |
| 6 | Religion | | | 1. Catholic 2. Muslim 3. Others specify……… |
| 7 | Marital status (**Tick one appropriate option**) | | | 1. never married (single) 2. married 3. widow 4. divorced 5. Others specify |
| 8 | Highest level of education/school completed (**Tick one appropriate option**) | | 1. No formal education 2. Primary level 3. Ordinary level of education 4. Certificate after completion of O level 5. Advanced level of Education 6. Tertiary level of Education 7. University level of Education | |
| 9 | If married or cohabiting, what is your spouse’s level of education? | | 1. No formal education 2. Primary level 3. Ordinary level of education 4. Certificate after completion of O level 5. Advanced level of Education 6. Tertiary level of Education 7. University level of Education | |
| 10 | What is your Current employment status? | | 1. Unemployed  2. Employed formally (specify title/cadre) ……………………………………  3. Other (specify…………………………… | |
| 11 | Name of the nearest health facility/hospital from participant’s home. | |  | |
| 12 | Level of that nearest health facility to participant’s home. | | 1. Health Centre III  2. Health Centre IV  3. Hospital (District/Faith based)  4. Regional Referral or Equivalent Faith based  5. National Referral hospital | |
| 13 | How far is this facility from your home (approximate KM as estimated by patient)? | |  | |

**SECTION 2: KNOWLEDGE & PERCEPTIONS ON BREAST CANCER**

14. Here are a list of things that I am going to read out to you. Please tell me, which of the following you think causes breast cancer? (**Please tick as appropriate**)

| **No.** | **Questions and filters** | | **Response** |
| --- | --- | --- | --- |
|  | Having breast cancer previously | | 1. No 2. Yes 3. Don’t know |
|  | Drinking more than 1 bottle of beer or 1 glass of other types of alcohol per day | | 1. No 2. Yes   777.Don’t know |
|  | Not breastfeeding **(ONLY FOR FEMALE RESPONDENTS)** | | 1. No 2. Yes   777. Don’t know |
| d) | Using hormone replacement therapy**(ONLY FOR FEMALE RESPONDENTS)**  [Explanation]: As women get older their hormone levels become lower and their periods stop. They may experience hot flushes and other discomforts. The medication to help with these discomforts is called hormone replacement therapy | 1. No 2. Yes   777. Don’t know | |
| e) | Wearing a tight bra **(ONLY FOR FEMALE RESPONDENTS)** | 1. No 2. Yes   777. Don’t know | |
| f) | Using hormonal family planning methods (e.g. the pill, injectable contraceptives and implants)  **(ONLY FOR FEMALE RESPONDENTS)** | 1. No 2. Yes   777. Don’t know | |
| g) | Being overweight | 1. No 2. Yes   777. Don’t know | |
| h) | Wearing a bra all the time, including at night when sleeping**(ONLY FOR FEMALE RESPONDENTS)** | 1. No 2. Yes   777. Don’t know | |
| i) | Having a family member with breast cancer | 1. No 2. Yes   777. Don’t know | |
| j) | Putting money in one’s bra  **(ONLY FOR FEMALE RESPONDENTS)** | 1. No 2. Yes   777. Don’t know | |
| k) | Having the first child after the age of 30 years  **(ONLY FOR FEMALE RESPONDENTS)** | | 1. No 2. Yes   777. Don’t know |
| l) | Bewitched/witchcraft/evil spirits | | 1. No 2. Yes   777. Don’t know |
| m) | Starting your periods **early**, before the age of 11 years**(ONLY FOR FEMALE RESPONDENTS)** | | 1. No 2. Yes   777. Don’t know |
| n) | Doing little physical activity or manual labour | | 1. No 2. Yes   777. Don’t know |
| o) | Aging/growing old | | 1. No 2. Yes |
|  |  |  | 777. Don’t know |
| p) | Putting a mobile phone in one’s bra  **(ONLY FOR FEMALE RESPONDENTS)** | | 1. No 2. Yes   777. Don’t know |
| q) | Not delivering any child at all  **(ONLY FOR FEMALE RESPONDENTS)** | | 1. No 2. Yes 3. Don’t know |
| r) | Having menopause **late**, after the age of 55 years  [Explanation]: This is when a woman’s period stops permanently **(ONLY FOR FEMALE RESPONDENTS)** | | 1. No 2. Yes   777. Don’t know |
| s) | Being exposed to dirty air or water | | 1. No 2. Yes   777. Don’t know |

15. Here are a list of things that I am going to read out to you that may be symptomsof breast cancer. Please tell me, which of the following you think are the signs or symptoms of breast cancer? (**Please tick as appropriate**)

| **No.** | | **Questions and filters** | | **Response** |
| --- | --- | --- | --- | --- |
|  | | A change in the position of the nipple  [Explanation]: such as pointing up or down or in a different direction to normal | | 1. No 2. Yes   777. Don’t know |
|  | | Pulling in of the nipple  [Explanation]: Where the nipple no longer points outwards but into the breast. | | 1. No 2. Yes 3. Don’t know |
|  | | A change in the shape of the nipple, not when pregnant or breast feeding | | 1. No 2. Yes 3. Don’t know |
|  | | Discharge from the nipple, not when pregnant or breast feeding | | 1. No |
|  |  |  |  | 1. Yes 2. Don’t know |
|  | | Bleeding from the nipple | | 1. No 2. Yes 3. Don’t know |
|  | | Pain in one or both breasts, not when pregnant or menstruating | | 1. No 2. Yes 3. Don’t know |
|  | | A lump or thickening in the breast | | 1. No 2. Yes 3. Don’t know |
|  | | Puckering or dimpling of the breast skin  [Explanation]: like a dent or orange peel appearance of the skin | | 1. No 2. Yes 3. Don’t know |
|  | | A change in the size of the breast, not when pregnant or breast feeding | | 1. No 2. Yes 3. Don’t know |
|  | | A lump or thickening under the armpit/under arm | | 1. No 2. Yes 3. Don’t know |
|  | | Pain in the armpit/under arm | | 1. No 2. Yes 3. Don’t know |
| 16. | Who should get tested or screened for breast cancer with x-ray of breast (Mammography)? (**Please tick only one that applies best. Do not read out the responses to the participant**) | | 1. Any female 2. Married women 3. Unmarried women 4. Sex workers 5. Any woman aged 49 years and above 6. Other………. | |
| 17. | Prior to being diagnosed with breast cancer, had you ever heard about this disease? | | 1. No 2. Yes | |
| 18. | If yes, where did you hear this from (source of information)? (**Tick all that applies – Do not read out responses to participant**) | | 1. Newspaper 2. Television 3. Radio 4. Family member/Relative 5. Health workers 6. Friends 7. Other…….. | |
| 19 | Breast cancer can be treated by the following methods (**please tick all that apply. Read out responses to participant**). | | 1. Surgery 2. Traditional medicine 3. Spiritual intervention/Prayers 4. Radiotherapy/Chemotherapy 5. Do Not know 6. Other……… | |
| 20. | Breast cancer can be treated and cured (**Tick only one that applies. Read out responses to participant**). | | 1. When detected early 2. At any stage 3. Other (please specify) ……   777. Do not Know | |
| 21. | Breast Cancer will always lead to death once detected in a woman irrespective of treatment. | | 1. No 2. Yes   777. Do not Know | |

| **SECTION 3: PRESENCE OF BREAST CANCER RISK FACTORS**  Thank you for sharing the above information. I would like to now ask you a few questions concerning your health care journey until presenting at the UCI. | | |
| --- | --- | --- |
| **22.** | Do you know any of your family members who has had breast cancer now or in the past? | 1. No 2. Yes   777. Do not Know |
| **23.** | If yes, who of your family member? | 1. Sister 2. Daughter 3. Mother 4. Aunt 5. Other (Specify)………. |
| **24.** | At what age did you start your menstrual periods?**(ONLY FOR FEMALE RESPONDENTS)** | 1. …………. Years 2. Do not remember |
| **25.** | Have your menstrual periods stopped for life (menopause)?  **(ONLY FOR FEMALE RESPONDENTS)** | 1. No 2. Yes |
| **26.** | If yes, at what age did you stop to have the blood flow?  **(ONLY FOR FEMALE RESPONDENTS)** | 1. ………years 2. Do not remember |
| **27.** | How many biological children have you had? | 1. No children 2. ……… Children (alive and dead) |
| **28.** | Are you using or ever used any hormonal contraceptives (family planning) before?**(ONLY FOR FEMALE RESPONDENTS)** | 1. No 2. Yes |
| **29.** | If yes to question **28**, how many years have you used them in total duration?  **(ONLY FOR FEMALE RESPONDENTS)** |  |
| **30.** | Has a health worker; nurse/medical doctor ever told you in the past whether you have……. (**Read out responses to participant and tick all that applies**). | 1. Cardiac/Heart problem 2. HIV/AIDS 3. Diabetes 4. Hypertension/High blood pressure 5. Other Cancer……………. 6. Sexually Transmitted Infection (STI) 7. Other diseases (Specify….) |
| **SECTION 4: FACTORS ON THE CARE PATHWAYS** | | |
| **31.** | When did you first notice the signs/symptoms of the cancer you have (**use calendar landmark approach to establish month & year**)? | ……. /………. (Month/Year) |
| **32.** | What were the first symptoms you noticed **(please tick all that apply**)? | 1. Pain in the breast 2. Lump or swelling in the breast 3. Discharge from the nipple 4. Ulcer or wound on the breast 5. Swelling under the arm pit 6. Difficulty in breathing 7. Pain on the back 8. Wounds on the breast 9. Swelling on the breast 10. Others…………………… |
| **33.** | At the beginning of this illness, what did you think was causing the above signs/symptoms? (**Please tick all responses said by participant. Do not read out any responses**). | 1. Trauma or injury to the breast 2. Thought it was something serious 3. Thought it wasn’t anything serious 4. TB of the breast 5. Bewitched/Witchcraft 6. Infections of the breast 7. Cancer of the breast 8. Other……………………… |
| **34.** | On noticing these changes (symptoms of this illness), whom did you first discuss them with? (**Tick only one response. Do not read out any responses**). | 1. Health worker at health facility 2. Relative (Mother/Father/Husband/Other) …………... **(tick/specify the one which is applicable)** 3. Traditional Healer 4. Spiritual Leader 5. Others specify……………… |
| **35.** | After you noticed these changes, how long did it take you to visit the health facility/health worker? | 1. within the first 2 weeks of noticing the symptoms 2. About a month 3. About 3 months 4. 6 months 5. 6 to 12 months 6. More than12 months |
| **36** | Number of times visited the healthy facility before referral | 1. Once 2. Twice 3. Thrice 4. Others specify………… |
| **37** | Number of times visited the referring facility | 1. Once 2. Twice 3. Thrice   Others specify………… |
| **38.** | How long did it take for you to visit the UCI from the time you were referred? | 1. Within the first 2 weeks of referral. 2. After about a month or two from date of referral. 3. After about 3 months to six months from time of referral. 4. After more than 6 months but less than a year from time of referral. 5. After more than a year (>12 months) from time of referral. |
| **39** | What was the reason for taking that period of time before reporting to the UCI? (**Tick all that the participant mentions. Please do not read out any responses**). | 1. Busy work schedule 2. Lack of finances/No money for transport and tests at the UCI 3. Was still using treatment from the referring/other hospital 4. Still using traditional medicine 5. Was still using Prayers/ Spiritual healers 6. Fear of the Cancer therapy (chemotherapy/Radiotherapy) 7. Other specify…………… |
| **40.** | What Cancer treatment have you received so far? (**Tick all that apply. Please read out responses**) | 1. Surgery 2. Chemotherapy 3. Radiotherapy 4. Other specify…………… |
| **41.** | How long did it take to start the cancer treatment from the time of histology diagnosis? |  |
| **42.** | What was the reason for that time taken? | 1. Looking for finances for treatment 2. Discussing with family members 3. Was still using traditional treatment 4. The doctors had not yet told me to start the cancer treatment 5. The Radiotherapy machine was not working 6. Other specify…………… |
| **43.** | Currently how are you able to cater for the medical bills? (**Please tick all that apply. Do not read out responses but probe to get as many responses as applicable**). | 1. Using my salary/employed 2. From my business 3. Family/Relatives contributions 4. Husband 5. Charity/NGO group 6. Other specify……………… |

**We have come to the end of this interview.**

**Thank you for participating. Your responses are very useful to us.**

## **Appendix V: Data abstraction tool (to be completed from patients’ medical file)**

**5.1: Dates regarding important events on pathway to care**

|  |  | **DATE (**DD/MM/YY) | **Name of facility** |
| --- | --- | --- | --- |
| 44 | Date of first visit to formal health facility since onset of symptom - month & year (as reported by the patient, while using the calendar landmark approach) |  |  |
| 45 | Date of Fine needle aspiration (as on referral form) |  |  |
| 46 | Date of Biopsy of breast (if applicable) |  |  |
| 47 | Date of Surgery (if applicable) |  |  |
| 48 | Date of Referral to UCI |  |  |
| 49 | Name of Referring Health facility |  |  |
| 50 | Date first seen at Referring Health facility |  |  |
| 51 | Name of Facility where Biopsy was done (as on histology Form) |  |  |
| 52 | Date of Biopsy as on the histology form |  |  |
| 53 | Date of breast cancer diagnosis (as on histology form) |  |  |
| 54 | Date first seen at the UCI |  |  |
| 55 | Date of first Chemotherapy Cycle |  |  |
| 56 | Date of first Radiotherapy Cycle |  |  |
| 57 | Date of most recent review at the UCI before current visit |  |  |

| **5.2 TUMOR CHARACTERISTICS** | | |
| --- | --- | --- |
| 58. | Stage of breast cancer at diagnosis | 1. IA 2. IB 3. II 4. III 5. IV 6. Metastatic disease (distance spread reported) |
| 59. | TNM staging system |  |
| 60. | Cancer Histology Type | 1. Ductal carcinoma in Situ 2. Invasive ductal carcinoma 3. Invasive lobular carcinoma 4. Medullary carcinomas 5. Papillary carcinomas 6. Invasive tubular carcinomas 7. Inflammatory breast cancer 8. Others, specify: ……… |
| 61. | Hormonal receptor status   1. Estrogen receptor | 1. Negative 2. Positive |
|  | 1. Progesterone receptor | 1. Negative 2. Positive |
|  | (c) HER2/new status | 1. Negative 2. I Positive |
| **5.3: ANTHROPOMETRY** | | |
| 62. | a) Weight at UCI……………………………………………………..  b) Height at UCI……………………………………………………….  c) BSA………………………………………………………….  d) BMI………………………………………………………… | |
| Name of Interviewer………………………………………………………………  Signature of Interviewer…………………………………………………………..  Date of Interview…………………………………………………………………. | | |
